# Unit‐Level Burnout Dimensions and Patient Experience: A Multilevel Study of Aggregated Contextual Strain in a Southeast European Health System

**DOI:** 10.1155/nrp/7626110

**Published:** 2026-10-25

**Authors:** Edona Haxhija, Drita Kruja

**Affiliations:** ^1^ European University of Tirana Tirana Albania uet.edu.al; ^2^ Department of Nursing Faculty of Nursing AAB College 10000 Prishtina Kosovo aab-edu.net; ^3^ Center for Sustainable Development Faculty of Economy, Business and Development European University of Tirana Tirana Albania uet.edu.al

**Keywords:** burnout, HCAHPS, MBI, multilevel analysis, nurse well-being, patient experience

## Abstract

**Purpose:**

To examine whether aggregated unit‐level nurse burnout is associated with patient‐reported experience across hospital units using a multilevel framework, conceptualizing burnout as aggregated contextual strain rather than homogeneous unit climate.

**Methods:**

A cross‐sectional multilevel study was conducted across 11 regional public hospitals in Albania (October 2024–July 2025). Nurse burnout was assessed using the Maslach Burnout Inventory among 2000 nurses, and patient experience was measured with HCAHPS among 679 patients. Burnout scores were aggregated at the clinical‐unit level. Multilevel linear modeling with patients nested within units examined associations between emotional exhaustion, depersonalization, personal accomplishment, and patient experience. Cluster‐robust standard errors and leave‐one‐unit‐out sensitivity analyses were conducted to assess estimate stability.

**Results:**

Patient experience varied significantly between units (ICC = 0.229). In the multilevel model, higher unit‐level emotional exhaustion and depersonalization were both associated with lower patient experience. Under cluster‐robust estimation (t‐distribution, df = 9), depersonalization remained statistically significant (robust *p* < 0.001), and emotional exhaustion remained significant (robust *p* = 0.028), whereas personal accomplishment was not significant (robust *p* = 0.187). Leave‐one‐cluster‐out and exact wild‐cluster‐bootstrap sensitivity analyses confirmed depersonalization as the most robust association across all specifications (bootstrap *p* = 0.003), whereas emotional exhaustion, while directionally consistent, was only borderline under the most conservative correction (exact wild‐bootstrap *p* = 0.052), underscoring the statistical fragility inherent to a 10‐cluster design.

**Conclusion:**

Aggregated unit‐level depersonalization, and to a lesser and more fragile extent emotional exhaustion, may be relevant to patient‐centered care, but the strength and consistency of these unit‐level estimates are constrained by the small number of analytical clusters and by heterogeneity in how those clusters were formed across hospitals. Given the modest number of clinical units, findings should be interpreted as exploratory contextual associations. These findings support further investigation of workload support and team‐level resources as potential targets for improving nurse well‐being and patient‐centered care.

## 1. Introduction

Hospitals are human‐centered environments where the quality of care is shaped not only by clinical procedures but also by the emotional presence and communication of healthcare professionals [1, 2]. Nurses, who maintain the most sustained contact with patients, play a central role in shaping how care is experienced at the bedside, and their engagement and attentiveness are closely linked to positive patient reports [3, 4]. As health systems increasingly prioritize patient‐centered metrics, the relational dimension of care has become a core indicator of institutional performance and accountability. This study examines all three Maslach burnout dimensions—emotional exhaustion, depersonalization, and personal accomplishment—as aggregated unit‐level indicators, without presupposing in advance which dimension would show the strongest or most statistically stable association with patient experience; that question is treated as empirical rather than assumed.

Nurse burnout has emerged as a global concern amid increasing workload pressures, staffing shortages, and systemic demands [5]. Recent global syntheses estimate substantial levels of burnout across the health workforce, reinforcing the scale and persistence of this challenge across healthcare systems [6]. Defined by emotional exhaustion, depersonalization, and reduced personal accomplishment, burnout may affect the relational dimension of care by limiting energy, empathy, and interpersonal engagement with patients [7, 8]. Although typically conceptualized as an individual‐level phenomenon, burnout may also reflect shared exposure to demanding work environments within clinical units, potentially shaping teamwork, communication, and the relational tone of care across these settings. Rather than assuming strong within‐unit consensus, the present study conceptualizes aggregated burnout as aggregated contextual strain, defined as average levels of work‐environment pressure observable within clinical units without implying homogeneous shared perceptions. Empirical evidence linking aggregated contextual strain at the unit level to patient‐reported experience remains limited, particularly outside Western Europe and North America.

In Albania, recent studies report moderate to high levels of burnout among nurses and identify workplace conditions, demographic factors, and structural constraints as key contributors [9–11]. Other national and regional research highlights the influence of job satisfaction, technical work conditions, and organizational context on nursing performance and care quality [12], while broader analyses emphasize the interplay between workforce factors and care outcomes [13]. Despite this emerging evidence, little is known about whether variation in aggregated contextual strain across clinical units corresponds to measurable differences in patient‐reported experience within the Albanian hospital context.

Patient experience, commonly assessed using instruments such as the Hospital Consumer Assessment of Healthcare Providers and Systems (HCAHPS), has become a key indicator of healthcare quality and institutional performance [14]. Patients’ perceptions of communication, responsiveness, and respect are strongly shaped by everyday interactions with nurses and are increasingly used to evaluate the accountability and effectiveness of healthcare services [4, 14]. Understanding how workforce well‐being is reflected in these perceptions is therefore essential for interpreting differences in care quality across clinical environments and for informing organizational improvement efforts.

In this study, burnout is measured using the Maslach Burnout Inventory (MBI), which captures emotional exhaustion, depersonalization, and reduced personal accomplishment [15]. Rather than conceptualizing burnout as either purely individual or as a cohesive unit‐level climate, the study treats aggregated burnout scores as indicators of aggregated contextual strain. By examining whether variation in aggregated contextual strain within clinical units corresponds with differences in patient‐reported experience, the study adopts a cautious multilevel perspective that considers burnout not only as an individual phenomenon but also as a potential contextual feature of clinical environments. A multilevel framework is therefore used to examine how patient experience varies across units in relation to aggregated contextual strain, allowing simultaneous consideration of individual patient responses and shared clinical‐unit conditions [16, 17].

Although conducted within the Albanian public healthcare system, the study offers contextually grounded evidence that contributes to the broader international discussion on nurse well‐being and healthcare quality [5, 18]. Differences in staffing structures, governance, and resource distribution across health systems warrant cautious interpretation of generalizability. By adopting a multilevel perspective, the study extends burnout research to a Southeast European context and provides exploratory evidence linking aggregated contextual strain at the unit level to patient‐reported care quality. Given methodological challenges associated with multilevel models based on small cluster counts, the present study incorporates conservative estimation strategies and sensitivity analyses to strengthen contextual inference.

## 2. Literature Review

Contemporary research on healthcare quality increasingly emphasizes patient experience alongside clinical indicators [4, 19]. Patient experience captures how individuals interpret communication, responsiveness, and emotional support during hospitalization, and instruments such as HCAHPS are widely integrated into quality‐monitoring and performance frameworks [14, 20]. Because nurses are consistently present inpatient care processes, their communication practices and engagement strongly influence how care is perceived [21, 22]. Patient experience has been linked not only to satisfaction but also to adherence, clinical outcomes, and institutional trust, underscoring its systemic relevance beyond individual satisfaction metrics [18, 23].

Within this context, nurse burnout has attracted sustained attention. Defined as a multidimensional construct encompassing emotional exhaustion, depersonalization, and reduced personal accomplishment [24, 25], burnout may influence engagement with patients and the relational aspects of care [5, 26]. Empirical evidence links burnout to reduced patient‐centered communication and relational engagement in clinical settings [27]. A substantial body of research links burnout to adverse workforce outcomes, including turnover intention, job dissatisfaction, and increased risk of clinical error [28, 29]. More recent research extends these associations to patient‐reported outcomes, suggesting that staff strain may be reflected in patient perceptions of care; however, reported effect sizes vary across settings and methodological approaches, indicating potential contextual heterogeneity [18, 21, 30].

This variability has prompted growing recognition that burnout may reflect not only individual psychological strain but also shared organizational conditions within clinical units. Nurses working in the same unit are often exposed to similar workload intensity, staffing patterns, and leadership climates, potentially generating collective strain patterns that influence teamwork and communication [1, 2, 8]. Evidence suggests that higher collective burnout may correspond with care omissions and less favorable patient evaluations, supporting the interpretation of burnout as a contextual work‐environment signal rather than solely an individual attribute [2, 21, 31].

Examining such relationships requires attention to organizational structure. Patients are nested within clinical units, and outcomes may reflect both individual encounters and shared contextual conditions [32, 33]. Multilevel analytical approaches enable simultaneous examination of these layers and allow assessment of whether between‐unit variation in patient experience corresponds to aggregated burnout levels [34, 35]. By accounting for clustering within clinical environments, these approaches provide a more precise understanding of how shared working conditions may shape patient perceptions of care.

Although several studies have employed multilevel designs [36, 37], evidence remains limited outside North America and Western Europe. Hospital‐level analyses may obscure meaningful intrahospital differences in workload, staffing, and team climate that shape patient perceptions [38, 39]. As a result, we still know relatively little about how shared strain within individual clinical units becomes reflected in patients’ reported care experiences. These gaps are particularly relevant in healthcare systems facing workforce shortages and resource constraints, where shared burnout conditions may be more pronounced and unevenly distributed across units [5, 18]. By investigating unit‐level burnout and patient‐reported experience within a multilevel framework, the present study contributes contextually grounded evidence from a Southeast European setting that remains underrepresented in the existing literature and clarifies whether aggregated contextual strain corresponds with systematic variation in patient‐reported care quality.

## 3. Theoretical Framework and Hypotheses

This study is guided by four complementary theoretical frameworks that clarify the development of nurse burnout in clinical settings and its potential impact on patient experience. Rather than treating burnout solely as an individual psychological state, the present framework integrates Maslach’s multidimensional theory of burnout [15], the Job Demands–Resources (JD–R) model [40], Donabedian’s model [41], and a multilevel systems perspective [42], to situate burnout within broader organizational and relational processes. These frameworks provide a coherent foundation for examining how aggregated contextual strain within clinical units may be reflected in patient‐reported care experiences. Together, these frameworks allow a more explicit conceptual linkage between work‐environment strain, burnout dimensions as process‐level responses, and patient experience as an outcome reflecting relational aspects of care.

Within this integrated perspective, burnout is understood not only as an individual response to occupational stress but also as a patterned manifestation of shared working conditions within clinical units, consistent with the study’s framing of aggregated contextual strain.

### 3.1. Maslach’s Multidimensional Theory of Burnout

Maslach’s conceptualization of burnout describes a syndrome comprising emotional exhaustion, depersonalization, and reduced personal accomplishment [7]. Each dimension reflects a different way in which prolonged occupational strain may affect professional functioning. Emotional exhaustion captures the depletion of emotional resources, depersonalization reflects distancing from patients, and reduced personal accomplishment relates to diminished professional efficacy. In nursing settings, these dimensions are closely linked to the relational aspects of care. Sustained emotional strain may limit attentiveness and responsiveness, while detachment from patients may weaken communication and empathy. Reduced professional efficacy may further influence motivation and engagement in patient‐centered care [5, 21, 24]. From this perspective, average levels of burnout within units may constitute aggregated contextual strain capable of shaping patient perceptions, without implying uniform staff agreement. While the three dimensions are theoretically interconnected, each may plausibly be reflected in patient experience through a different relational pathway: emotional exhaustion through reduced attentiveness and energy, depersonalization through distancing and diminished empathy, and personal accomplishment through reduced engagement and motivation. Which dimension proves most directly and robustly associated with patient‐reported experience is treated as an empirical question in this study rather than a theoretical presupposition.

### 3.2. JD–R Model

The JD–R model provides a complementary explanation by linking burnout to the balance between job demands and available resources [40]. High workloads, staffing shortages, and time pressure can elevate job demands, while teamwork, leadership support, and adequate staffing function as resources that may buffer strain. Variation in these conditions across hospital units may lead to differences in burnout levels within the same institution. When job demands consistently exceed available resources, sustained strain may influence communication, responsiveness, and engagement in patient interaction [1, 2, 18]. This framework supports interpreting burnout at the unit level as aggregated contextual strain reflecting shared exposure to demanding working conditions rather than solely individual stress responses.

### 3.3. Donabedian’s Structure–Process–Outcome Model

Donabedian’s model offers a useful framework for linking workplace conditions with patient outcomes [41]. In this case, structural conditions include things like the number of staff and the amount of work that needs to be done. Within this perspective, burnout may be understood as a process‐level mechanism through which structural pressures are translated into observable relational behaviors in everyday care encounters. Patient experience, in turn, is a result that these processes may influence [18]. Within this framework, aggregated contextual strain may function as a process‐level mechanism through which structural pressures influence patient‐reported outcomes, without assuming the existence of a fully homogeneous unit climate. In this sense, contextual strain at the unit level helps explain how structural constraints become reflected in patient‐reported care quality, without assuming that all staff members experience these pressures in the same way. In this way, burnout is positioned not only as an individual condition but as a process‐level pathway linking structural constraints to patient‐perceived care quality.

### 3.4. Multilevel Systems Perspective

Hospital care operates within hierarchical systems in which patients are nested within clinical units and units within hospitals [42]. Because staff within the same unit are exposed to similar organizational demands and resources, patterns of strain may emerge at the unit level and influence communication, teamwork, and responsiveness to patients [21, 33].

A multilevel perspective allows examination of whether variation in patient experience reflects individual patient characteristics or contextual conditions observable at the unit level. In this study, burnout is examined at the unit level to capture aggregated contextual strain within shared working environments, while explicitly acknowledging that within‐unit agreement may vary across dimensions.

### 3.5. Conceptual Model

Drawing on these integrated theoretical perspectives, a conceptual model was developed to illustrate how nurse burnout at the unit level and its core dimensions may influence patient‐reported experience across clinical units (Figure 1). The model assumes that average burnout levels within units may shape nurse–patient interaction and patient perceptions of care quality, without implying cohesive collective climate effects. Figure 1 presents the conceptual model.

**FIGURE 1:**
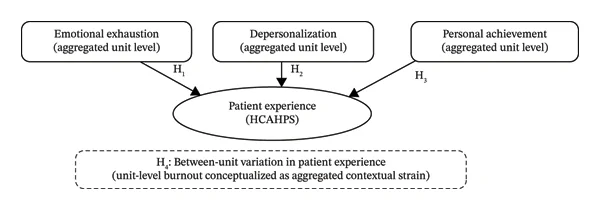
Conceptual model of unit‐level nurse burnout and patient experience. Burnout dimensions are modeled as indicators of aggregated contextual strain at the clinical‐unit level, reflecting average work‐environment pressure rather than homogeneous team‐level consensus.

The framework therefore considers the following:

• Unit‐level burnout dimensions (emotional exhaustion, depersonalization, personal accomplishment)
• Aggregated contextual strain reflected in average burnout levels within units
• Patient‐reported outcomes measured through HCAHPS scores

These elements interact within a multilevel structure in which nurses and patients are nested within the same clinical units. Based on the integrated theoretical framework and the proposed conceptual model, the following hypotheses were formulated to examine the relationship between unit‐level nurse burnout dimensions and patient‐reported experience across clinical units.

Consistent with Maslach’s multidimensional model, the three burnout dimensions are hypothesized separately (H_1_–H_3_) with equal a priori standing; no dimension is assumed in advance to be the primary or most robust contextual indicator, and the Results and Discussion report which dimension(s) proved statistically robust once small‐cluster corrections were applied.

 H_1_: Higher unit‐level emotional exhaustion is associated with lower patient experience (HCAHPS) scores across clinical units *(Maslach theory; JD–R model; Donabedian model).*
 H_2_: Higher unit‐level depersonalization is associated with lower patient experience (HCAHPS) scores across clinical units *(Maslach theory; Donabedian model; multilevel theory).*
 H_3_: Lower unit‐level personal accomplishment is associated with lower patient experience (HCAHPS) scores across clinical units *(Maslach theory; JD–R model).*
 H_4_: Patient experience varies between clinical units, reflecting appreciable between‐unit variation in patient‐reported outcomes and the potential contribution of contextual conditions observable at the unit level *(multilevel theory; Donabedian model).*

To facilitate interpretation of the findings, an interpretative model is presented (Figure 2), illustrating the potential pathway through which unit‐level burnout may be reflected in patient‐reported experience. The figure uses emotional exhaustion as an illustrative example dimension for this generic pathway; the same interpretive logic applies to depersonalization, which the Results and Discussion identify as the more statistically robust of the two significant dimensions once small‐cluster corrections are applied. Within this framework, sustained work‐environment strain (whichever dimension predominates) is conceptualized as a contextual indicator that may influence communication, attentiveness, and responsiveness in nurse–patient interactions. These relational processes, in turn, may shape how patients perceive the quality of care. This simplified pathway does not imply causality but provides a conceptual interpretation of how aggregated contextual strain may become observable in patient‐reported outcomes.

**FIGURE 2:**
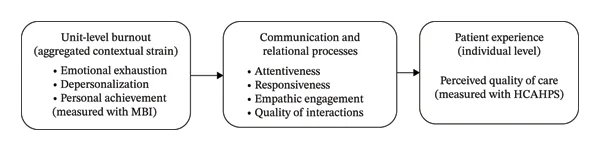
Interpretative model linking unit‐level burnout and patient experience. Note. This model illustrates a potential pathway based on theoretical integration of Maslach’s burnout theory, the JD–R model, and Donabedian’s structure–process–outcome framework.

## 4. Materials and Methods

### 4.1. Research Design

This study employed a cross‐sectional multilevel design to examine the association between nurse burnout and patient‐reported experience across hospital units. Data were collected between October 2024 and July 2025 from 11 regional public hospitals in Albania using two standardized instruments: the MBI for nurses [15] and the HCAHPS survey for patients [14].

The study focused on inpatient clinical units, including emergency, surgery, maternity, internal medicine, intensive care, neurology, cardiology, pneumology, otorhinolaryngology, and infectious diseases. The clinical unit served as the primary contextual level because nurses and patients shared the same care environment. The hierarchical structure of the data, with patients nested within clinical units, enabled examination of whether unit‐level burnout, conceptualized as aggregated contextual strain, was associated with patient‐reported experience. Aggregation was used to represent average work‐environment strain within units without assuming homogeneous staff perceptions. The unit level was therefore treated as the primary contextual layer (Level 2), while individual patient responses were modeled at Level 1.

The consolidation of functionally similar clinical units across hospitals into 10 analytical clusters is acknowledged as a methodological limitation rather than a preferred design choice. Because an intensive care unit in one hospital may differ substantially from an intensive care unit in another hospital in staffing, leadership, and workload, this clustering approach may increase within‐cluster heterogeneity and does not fully correspond to the concept of a shared contextual environment. The degree of cross‐hospital mixing within each cluster is documented directly in Supporting Table S1, which shows that clusters range from drawing on a single hospital to pooling patients from all 11 hospitals. This heterogeneity is discussed further as a limitation in the Discussion and Limitations sections.

### 4.2. Measures

#### 4.2.1. Nurse Burnout

Nurse burnout was measured using the MBI [15], which assesses emotional exhaustion, depersonalization, and personal accomplishment.

Individual nurse scores were calculated following standard procedures and aggregated at the clinical‐unit level to represent aggregated contextual strain within each unit. Higher scores on emotional exhaustion and depersonalization indicate greater burnout, whereas lower scores on personal accomplishment reflect higher burnout. Aggregation allowed examination of whether average burnout levels within units corresponded with patient‐reported care experience, without assuming full within‐unit agreement.

#### 4.2.2. Patient Experience

Patient experience was assessed using an adapted HCAHPS questionnaire [14], based on the CMS instrument and administered for research purposes within the local context.

The adapted instrument included 17 items covering key domains of patient experience, including nurse communication (3 items), physician communication (3 items), responsiveness of hospital staff (2 items), pain management (2 items), communication about medications (1 skip‐logic screening item plus 2 substantive content items, scored only when applicable), and hospital environment (2 items), as well as a global hospital rating and recommendation item.

Most items were measured on a four‐point frequency scale ranging from 1 (“*never*”) to 4 (“*always*”), while the global hospital rating was measured on a 0–10 scale. The recommendation item used a four‐point scale.

The questionnaire was translated into Albanian using forward–backward translation. Two bilingual healthcare researchers translated the instrument, and a blinded translator performed back‐translation. Discrepancies were resolved by an expert panel of nursing faculty and clinicians to ensure semantic and contextual equivalence.

The adapted questionnaire was piloted with 20 hospitalized patients to assess clarity and feasibility only; this pilot was used exclusively for wording refinement and was not used for any psychometric estimation. Minor wording refinements were introduced without altering item structure or response scales. Construct validity was examined through exploratory and confirmatory factor analysis conducted on the full analytic sample of hospitalized patients (*N* = 679), not on the 20‐patient pilot sample. Because the questionnaire items are ordinal, polychoric correlations were used as input to the factor analyses, and the confirmatory model was estimated using a diagonally weighted least squares (DWLS) approach appropriate for ordinal indicators (see Section 5.3 for full EFA/CFA results and fit indices). The results supported a strong, dominant general patient‐experience factor rather than a set of clearly separated multidimensional domains; the factor structure is described in detail in Section 5.3.

To ensure comparability across items, all applicable items were rescaled to a common 1–4 metric, with higher scores indicating better patient experience. The composite patient‐experience score was calculated as the mean of all nonmissing items measured on the 1–4 scale. The global rating item (0–10) was linearly rescaled to the 1–4 metric prior to inclusion using the transformation rescaled = 1 + 0.3 × raw, ensuring the composite is bounded within the stated 1–4 range. The medication–communication domain includes only its two substantive items (Items 12 and 13); the conditional screening question (Item 11, “were you given a new medication, yes/no”) is a skip‐pattern item and is not included in domain or composite scoring. Nonapplicable responses (i.e., patients who did not receive a new medication during their stay) were treated as structurally missing and excluded from composite calculations; this affected Item 12 for 43 of 679 patients (6.3%; see Supporting Table S2).

Internal consistency of the final composite was assessed using Cronbach’s alpha (*α* = 0.94 for the 16‐item composite), indicating excellent reliability. Domain‐level alphas ranged from 0.63 (responsiveness of hospital staff, 2 items) to 0.99 (medication communication), with most domains above 0.85. The high overall alpha, combined with the factor structure reported in Section 5.3, likely reflects a single dominant general patient‐experience factor rather than conceptual overlap among clearly distinct domains.

A one‐unit increase in the patient‐experience score represents an increase of one point on the standardized composite scale, reflecting higher overall perceived quality of care across included domains. Unit‐level values represent aggregated mean patient‐experience scores within clinical units and are not directly comparable to externally benchmarked CMS performance metrics.

#### 4.2.3. Control Variables

Basic demographic information for nurses (age, gender, and education level) was collected and considered as potential covariates in the multilevel models to account for individual‐level differences. These variables were examined in preliminary model specifications to assess potential confounding effects on the association between unit‐level burnout and patient experience.

### 4.3. Questionnaire Design

Both instruments were administered in standardized formats and linguistically adapted to ensure clarity within the Albanian healthcare context. Details regarding translation, cultural adaptation, and pilot testing are described in the measures section.

The MBI was completed anonymously by nurses during work shifts. Patient‐experience data were collected through structured interviews prior to discharge. Only patients treated in units with nurse data were included to ensure contextual alignment. Full instruments are provided in the Appendix.

### 4.4. Sampling

The study included 11 regional public hospitals representing major inpatient providers within Albania’s public healthcare system. These hospitals reflect typical public‐sector conditions, including variability in staffing and workload across units.

Eligible participants included nurses working in inpatient clinical units and patients hospitalized in the same units during the study period. Patients younger than 18 years or those unable to provide informed consent were excluded. Nurses were excluded if they had incomplete MBI responses or were not directly involved in inpatient clinical care.

A total of 2633 eligible nurses were identified, of whom 2085 completed the MBI questionnaire. After excluding 85 nurses due to incomplete responses or lack of involvement in inpatient care, 2000 nurses were included in the unit‐level aggregation (response rate ≈ 76%) (see Figure 3).

**FIGURE 3:**
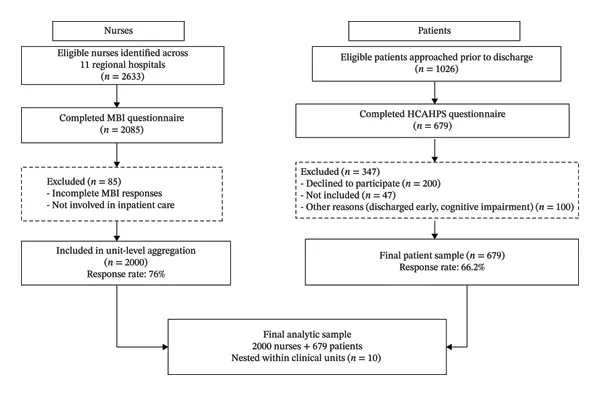
Participant flow diagram for nurses and patients.

Patient recruitment followed a consecutive sampling approach within the included clinical units. A total of 1026 eligible patients were approached prior to discharge, of whom 679 consented and completed the HCAHPS questionnaire, resulting in a response rate of approximately 66.2%. A total of 347 patients were excluded due to refusal (*n* = 200), ineligibility (*n* = 47), or other reasons such as early discharge or cognitive inability (*n* = 100). Participation rates varied modestly across hospitals, with no consistent pattern of nonresponse suggesting systematic bias (see Figure 3).

The distribution of eligible and participating nurses and patients across hospitals is presented in Table 1, providing transparency regarding cluster composition within the multilevel structure. Only patients treated in units with corresponding nurse data were included to ensure contextual alignment. The final analytic sample consisted of 2000 nurses and 679 patients.

**TABLE 1:** Distribution of eligible and participating nurses and patients across regional hospitals.

No.	Regional hospital	Eligible nurses (n)	Participating nurses (n)	Eligible patients (n)	Participating patients (n)
1	Shkodër	310	280	195	146
2	Kukës	170	140	40	21
3	Dibër	170	166	85	60
4	Lezhë	160	150	92	68
5	Durrës	359	200	120	95
6	Elbasan	290	200	80	50
7	Fier	232	200	85	50
8	Vlorë	412	200	90	57
9	Korçë	240	200	83	56
10	Gjirokastër	90	86	67	26
11	Berat	200	178	89	50
**Total**		**2633**	**2000**	**1026**	**679**

*Note:* Values are presented as eligible and participating participants. Participating nurses contributed to the estimation of unit‐level burnout indicators, while participating patients correspond to the final analytic sample used in the multilevel analysis. The bold values indicate the overall totals across all 11 regional hospitals: 2633 eligible nurses, 2000 participating nurses, 1026 eligible patients, and 679 participating patients.

Burnout indicators were aggregated at the clinical‐unit level to represent aggregated contextual strain within units, producing a hierarchical dataset with patients nested within clinical units. In the multilevel analysis, clinical units were grouped into 10 analytical clusters based on functional similarity (e.g., intensive care, surgery, internal medicine) across hospitals. This approach was selected to create functionally comparable Level‐2 groupings while avoiding very small hospital‐by‐unit cells. Because some analytical clusters pooled functionally similar units from multiple hospitals, these clusters should not be interpreted as homogeneous shared work environments. Modeling each hospital‐by‐unit combination separately was considered but not adopted, as further disaggregation would have resulted in a larger number of small clusters, potentially reducing the precision and stability of contextual estimates in a small‐cluster multilevel design.

Although the number of analytical clusters (*n* = 10 in the final multilevel analysis) was modest, each analytical cluster contained substantial numbers of nurses and patients, supporting relatively stable aggregation. Multilevel studies with limited cluster counts have been applied in health services research when clusters represent meaningful organizational contexts and within‐cluster sample sizes are substantial [39, 43]. Cluster‐level findings are interpreted conservatively as exploratory contextual associations emphasizing direction and consistency rather than precise contextual effect sizes.

Ethical approval was obtained from the relevant institutional review board, and informed consent was secured from all participants.

The comparability of the 10 analytical clusters used in the multilevel models—including the number of distinct hospitals contributing patients to each cluster—is documented in Supporting Table S1. Clusters range from drawing on a single hospital (Cluster 16, *n* = 21 patients) to pooling patients from all 11 hospitals (Clusters 1 and 4), meaning the 10 clusters do not represent a uniformly comparable level of cross‐hospital aggregation; this is discussed further as a limitation of the clustering approach in the Discussion and Limitations sections.

Full missing‐data documentation by item is provided in Supporting Table S2. Only one item across the entire 17‐item questionnaire had any missing responses: Item 12 (“before giving you any new medicine, how often did staff tell you what the medicine was for”), which is structurally conditional on Item 11 (“were you given any medicine you had not taken before”). All other items, and the corrected composite score itself, had 0% missingness.

### 4.5. Data Analysis

Descriptive statistics and preliminary data management were conducted in SPSS Version 27. Multilevel modeling (restricted maximum likelihood [REML] estimation of random‐intercept models), cluster‐robust inference with small‐cluster t‐based inference (df = *G* − 1 = 9), the exact wild‐cluster‐bootstrap procedure, and the polychoric correlation–based exploratory and confirmatory factor analyses were conducted in R Version 4.4.1 (R Core Team, 2024) using the packages lme4 (mixed‐effects models), clubSandwich (cluster‐robust variance estimation), lavaan (confirmatory factor analysis using the DWLS estimator for ordinal data), and fwildclusterboot (wild‐cluster‐bootstrap inference). Burnout was aggregated at the clinical‐unit level, while patient experience was retained at the individual level to reflect the hierarchical structure of the data. Unit‐level burnout indicators were conceptualized as aggregated contextual strain rather than homogeneous unit climate.

Pearson correlations were examined at the unit level, followed by multilevel linear modeling with patients (Level 1) nested within clinical units (Level 2). A null model was estimated to compute the intraclass correlation coefficient (ICC) and quantify between‐unit variance. Burnout dimensions were then entered as unit‐level predictors. Unit‐level predictors were grand‐mean centered prior to modeling to facilitate interpretation of the intercept and reduce potential multicollinearity. Variance inflation factors computed at the patient level (*n* = 679, treating each patient as an independent observation) were below 3; however, because emotional exhaustion, depersonalization, and personal accomplishment are Level‐2 (unit‐level) predictors that are constant within each cluster, patient‐level VIFs substantially understate the true collinearity at the level at which these coefficients are actually identified. Recomputed at the unit level (*n* = 10, the correct level of analysis for these predictors), VIFs were 6.17 (emotional exhaustion), 8.95 (depersonalization), and 2.38 (personal accomplishment) (see Supporting Table S3). This confirms nontrivial collinearity between emotional exhaustion and depersonalization at the unit level (consistent with their *r* = 0.87 intercorrelation, Table 2), which may contribute to coefficient instability in the sensitivity analyses and to the personal‐accomplishment sign reversal discussed below.

**TABLE 2:** Pearson correlations among unit‐level burnout dimensions and patient experience.

Variable	EE	DP	PA	HCAHPS
Emotional exhaustion (EE)	1	0.873	−0.321	−0.202
Depersonalization (DP)	0.873	1	−0.617	−0.168
Personal accomplishment (PA)	−0.321	−0.617	1	0.044
HCAHPS	−0.202	−0.168	0.044	1

*Note:* Values represent Pearson correlations at the unit level.

Although the number of Level‐2 analytical clusters (*n* = 10) was modest, each analytical cluster contained a substantial number of nurses and patients, supporting relatively stable aggregation. Given the small number of Level‐2 analytical clusters, contextual fixed‐effect estimates and standard errors were interpreted conservatively, consistent with methodological guidance for multilevel models based on limited cluster counts. Prior research indicates that models with few clusters may produce less precise contextual estimates [44, 45], with increased Type I error risk in simulation studies [46]. Recent methodological guidance suggests that exploratory multilevel estimates remain informative when cluster sizes are large and theoretically justified [47]. Findings are interpreted as contextual associations reflecting aggregated contextual strain rather than confirmatory causal effects [42].

Burnout dimensions were entered simultaneously as Level‐2 predictors. Random slopes were not estimated due to limited clusters, and effects were modeled as fixed across units. Cross‐level interactions were not specified.

Deviance statistics (−2 log‐likelihood) and variance components were obtained from the REML‐fitted random‐intercept models. Because the compared models differed in their fixed‐effects specification, REML deviance values were treated descriptively rather than as a formal likelihood‐based comparison of model fit. Cases with structurally missing item‐level responses were excluded from the affected item only (pairwise, not listwise), since only a single item (Item 12, 6.3% missing, structural) had any missing data; no patient had a missing composite score (see Supporting Table S2 for full missing‐data documentation). Statistical significance was set at *p* < 0.05, with cluster‐robust inference for fixed effects evaluated against a t‐distribution with df = *G* − 1 = 9 (*G* = number of Level‐2 clusters), consistent with small‐cluster correction guidance, rather than a normal‐theory (z) approximation. Because structural indicators (staffing ratios, workload intensity, leadership climate) were unavailable, aggregated burnout may partly reflect broader working conditions within units. Findings are therefore interpreted as associations between aggregated contextual strain and patient experience. Patient‐level covariates (age and length of stay) were examined in preliminary multilevel models because of their plausible associations with patient‐reported experience. Their inclusion did not materially alter the magnitude or direction of the unit‐level burnout coefficients; therefore, the more parsimonious model is presented as the primary specification.

Five Supporting tables provide additional diagnostic and sensitivity analysis detail supporting the results reported below: cross‐hospital cluster composition (Supporting Table S1), item‐level missing‐data documentation (Supporting Table S2), variance inflation factors at the correct unit level (Supporting Table S3), domain‐level reliability and CFA loadings (Supporting Table S4), and leave‐one‐cluster‐out sensitivity results for all three burnout dimensions (Supporting Table S5).

## 5. Results

### 5.1. Descriptive Statistics

The final analytical sample included 679 patients nested within 10 analytically constructed functional clusters. Descriptive statistics were examined at both the patient and analytical‐cluster levels (see Table 3). At the patient level, mean patient experience scores were moderate (*M* = 2.572, SD = 0.853; *N* = 679), using the corrected 1–4 composite described in Section 4.2.2.

**TABLE 3:** Descriptive statistics of analytical‐cluster‐level study variables (*n* = 10 analytical clusters).

Variable	Mean	SD	Min	Max
HCAHPS	2.702	0.475	2.175	3.765
Emotional exhaustion	18.33	2.03	16.00	21.38
Depersonalization	10.26	3.82	4.67	16.57
Personal accomplishment	29.79	3.78	25.33	36.13

At the analytical‐cluster level, Table 3 presents descriptive statistics for aggregated burnout indicators and mean patient‐experience scores (*n* = 10 analytical clusters). Mean HCAHPS scores were moderate (*M* = 2.702, SD = 0.475), indicating variability in patient experience across analytical clusters, with the corrected composite now bounded within its nominal 1–4 range (min = 2.175, max = 3.765; see Table 3). Emotional exhaustion (*M* = 18.33, SD = 2.03), depersonalization (*M* = 10.26, SD = 3.82), and personal accomplishment (*M* = 29.79, SD = 3.78) also varied across analytical clusters, with depersonalization showing the greatest dispersion.

These patterns indicate meaningful between‐cluster variability in burnout dimensions and patient experience, supporting further examination of contextual associations at the analytical‐cluster level.

Given the observed variability for depersonalization, additional distributional checks were conducted. Visual inspection and influence diagnostics indicated no substantial deviation from approximate normality or extreme cluster‐level outliers, suggesting that the observed variability reflects genuine between‐cluster differences rather than measurement inconsistencies.

### 5.2. Correlation Analysis

Pearson correlations were computed using aggregated unit‐level data (*n* = 10 clinical units) and the corrected HCAHPS composite. Results indicated that depersonalization was weakly to moderately negatively associated with patient experience (*r* = −0.17), emotional exhaustion showed a weak negative association (*r* = −0.20), and personal accomplishment showed a negligible positive association (*r* = 0.04); these modest bivariate correlations illustrate how sensitive small‐cluster correlation estimates are to correct composite scoring. Burnout dimensions were themselves strongly intercorrelated (EE–DP *r* = 0.87), consistent with Maslach’s theoretical model but also indicating limited discriminant separation between these two dimensions at the unit level in this sample; this intercorrelation, rather than the composite scoring itself, explains why EE and DP show similar signs and magnitudes in the regression model below.

Given the limited number of clusters, correlation coefficients should be interpreted as descriptive indicators of between‐unit patterns rather than inferential estimates, and the strong EE–DP intercorrelation should be kept in mind when interpreting the independent regression coefficients for each dimension in Table 4.

**TABLE 4:** Multilevel model predicting patient experience (conventional and cluster‐robust estimates).

Predictor	B	Model‐based SE	Robust 95% CI, *t* (9)	Robust SE	Robust *p*
Emotional exhaustion	−0.0221	0.0097	[−0.0411, −0.0032]	0.0084	0.028
Depersonalization	−0.0146	0.0018	[−0.0194, −0.0098]	0.0021	< 0.001
Personal accomplishment	−0.0187	0.0076	[−0.0483, 0.0109]	0.0131	0.187

### 5.3. Factor Structure and Internal Consistency of the Patient‐Experience Composite

To evaluate the underlying structure of the patient‐experience measure and confirm that construct‐validity analyses reflect the full analytic sample rather than the small pilot sample used for wording refinement, exploratory and confirmatory factor analyses were conducted on the full analytic sample (*N* = 679) using polychoric correlations among the 15 ordinal patient‐experience items (Items 1–10, 12–15, 17; the 0–10 global item was excluded from the factor model and analyzed separately, consistent with standard HCAHPS composite‐scoring conventions). The factor analyses were restricted to these 15 ordinal items, whereas the final patient‐experience composite contained 16 scored items after inclusion of the linearly rescaled 0–10 global hospital‐rating item. The global rating was therefore included in the composite score but excluded from the ordinal factor analyses.

An eigenvalue decomposition of the polychoric correlation matrix showed one dominant factor (eigenvalue = 10.08, accounting for 67.2% of total variance), with the second and third eigenvalues far smaller (1.25 and 1.22, 8.3% and 8.1%, respectively). A one‐factor confirmatory model was estimated for the 15 ordinal patient‐experience items using the DWLS estimator for ordinal data. Standardized loadings ranged from 0.71 to 0.87, with all items loading strongly on the general factor. Model‐fit indices were RMSEA = 0.072, CFI = 0.979, TLI = 0.976, and SRMR = 0.107 (*χ* ^2^ ≈ 405.5, df = 90, *N* = 679). CFI and TLI indicate excellent fit; RMSEA is in the acceptable range; SRMR is somewhat elevated, indicating modest residual covariation among items within the same conceptual domain beyond the general factor. These findings support a dominant general patient‐experience factor, although the elevated SRMR indicates some residual multidimensionality or local dependence beyond the general factor. This is consistent with the high overall Cronbach’s alpha (*α* = 0.94) and supports use of the overall composite as the primary outcome.

Domain‐level Cronbach’s alphas (raw 1–4 items) ranged from 0.63 (responsiveness of hospital staff, 2 items) to 0.99 (medication communication, corrected formula); full domain‐level alphas and one‐factor CFA loadings are reported in Supporting Table S4. Given the dominant single‐factor structure, the overall composite score is used as the primary outcome throughout the Results, consistent with the original analytic strategy, while the domain structure is presented descriptively rather than as evidence of separate validated subscales.

### 5.4. Null Model and Intraclass Correlation

A null multilevel model was estimated (REML, corrected HCAHPS composite) to assess between‐unit variation in patient experience. The between‐unit variance was 0.191, while the within‐unit variance was 0.643. The ICC was 0.229, indicating that 22.9% of the variance in patient experience occurred between clinical units.

This level of between‐unit variation supports the use of a multilevel framework and suggests that patient experience differs meaningfully across clinical environments. This finding supports H4 and justifies the inclusion of unit‐level predictors in subsequent models (see Table 5).

**TABLE 5:** Null‐model variance components and intraclass correlation.

Parameter	Estimate	SE
Between‐unit variance (intercept)	0.191	—
Within‐unit variance (residual)	0.643	—
Intraclass correlation (ICC)	0.229	—

### 5.5. Aggregation Diagnostics

To evaluate whether nurse burnout could be meaningfully treated as a unit‐level construct, aggregation diagnostics were examined using nurse‐level data. Intraclass correlations and within‐unit agreement indices were calculated for each burnout dimension prior to multilevel modeling.

Emotional exhaustion demonstrated modest clustering (ICC [1] = 0.07) and high reliability of aggregated means (ICC [2] = 0.91). Although within‐unit agreement was modest (mean rwg = 0.24), reliability of unit‐level means suggests emotional exhaustion can be interpreted as an approximate contextual feature of the clinical work environment (see Table 6).

**TABLE 6:** Aggregation diagnostics for nurse burnout dimensions.

Dimension	ICC (1)	ICC (2)	rwg mean	% ≥ 0.70
EE	0.07	0.91	0.24	10%
DP	0.01	0.56	−0.46	0%
PA	0.02	0.75	0.15	0%

Depersonalization showed minimal between‐unit variance (ICC [1] = 0.01), lower reliability (ICC [2] = 0.56), and very low within‐unit agreement (mean rwg = −0.46). It was therefore retained as an exploratory indicator of aggregated contextual strain rather than interpreted as a homogeneous or stable unit‐level climate construct (see Table 6).

Personal accomplishment demonstrated modest clustering (ICC [1] = 0.02) and acceptable reliability (ICC [2] = 0.75), though within‐unit agreement remained limited (mean rwg = 0.15). Although agreement indices were modest, the reliability of aggregated means supported cautious inclusion of this dimension as an approximate indicator of shared professional efficacy within units (see Table 6).

Emotional exhaustion most clearly reflected a structured contextual pattern at the unit level, whereas depersonalization and personal accomplishment showed greater heterogeneity. All aggregated indicators were retained for exploratory multilevel modeling to capture aggregated contextual strain within units. Findings should therefore be interpreted as exploratory signals requiring replication in larger multilevel samples.

Aggregation diagnostics suggest that burnout dimensions vary in the degree to which they reflect contextual conditions at the unit level; therefore, all unit‐level interpretations are presented conservatively.

### 5.6. Multilevel Model

A two‐level linear mixed‐effects random‐intercept model (REML) was estimated with patients nested within clinical units, using the corrected HCAHPS composite as the outcome. Burnout dimensions were entered simultaneously as unit‐level predictors of patient experience. Given the modest number of Level‐2 units (*n* = 10), both conventional model‐based and cluster‐robust (CR1 sandwich) standard errors are reported to provide a transparent assessment of statistical uncertainty. Because only 10 clusters were available, cluster‐robust inference was evaluated using a t‐distribution with df = *G* − 1 = 9 rather than a normal (z) approximation. Statistical inference for the fixed effects was based primarily on the cluster‐robust estimates.

Under cluster‐robust *t* (9) inference, higher unit‐level depersonalization was associated with lower patient experience (robust *p* < 0.001), and emotional exhaustion also showed a negative association (robust *p* = 0.028). Personal accomplishment (robust *p* = 0.187) was not statistically significant (see Table 4). Because emotional exhaustion and depersonalization were strongly intercorrelated at the unit level (*r* = 0.87; Table 2), and unit‐level VIFs were 6.17 and 8.95, respectively, their mutually adjusted coefficients should be interpreted cautiously because of nontrivial collinearity.

Under this primary *t* (9)‐based specification, both emotional exhaustion and depersonalization showed negative unit‐level associations with patient experience. However, the stability of these estimates differed in the subsequent small‐cluster sensitivity analyses: Depersonalization remained the more consistent association, whereas the emotional‐exhaustion estimate was more sensitive to the inferential method and cluster exclusions. Personal accomplishment showed a statistically nonsignificant adjusted association. Its coefficient was negative despite a negligible positive bivariate correlation with patient experience (*r* = 0.04). Verification of item coding and scale direction did not identify a coding error. Given the substantial shared variance among the burnout dimensions, this sign reversal may reflect statistical suppression or coefficient instability and is therefore treated as an exploratory finding rather than a substantive effect.

Sensitivity models additionally included patient‐level covariates (age and length of stay). Their inclusion did not materially change the magnitude or direction of the unit‐level burnout coefficients. Given the limited number of Level‐2 clusters and the study’s primary focus on unit‐level contextual associations, the more parsimonious specification is presented as the primary model, while the covariate‐adjusted models were used to assess robustness. Residual confounding from unmeasured patient‐ and unit‐level characteristics remains possible.

### 5.7. Leave‐One‐Cluster‐Out Sensitivity Analysis

Given the modest number of Level‐2 clusters (*n* = 10), all three burnout‐dimension coefficients were reestimated 10 times, each time excluding one cluster, with cluster‐robust standard errors and *t* (df = 8) inference (full results in Supporting Table S5). Depersonalization was the most stable: It remained statistically significant (*p* < 0.01) in all 10 leave‐one‐out exclusions. Emotional exhaustion remained negative and of similar magnitude in every exclusion (range: −0.015 to −0.026) but crossed the *p* < 0.05 threshold in only 6 of 10 exclusions. Personal accomplishment was never statistically significant in any exclusion. This pattern reinforces depersonalization, rather than emotional exhaustion, as the more robust unit‐level signal once small‐cluster fragility is taken into account, and is one reason for inference in this study, which is framed as exploratory rather than confirmatory.

As an additional small‐cluster robustness check, we computed wild‐cluster‐bootstrap *p*‐values (Rademacher weights, exact enumeration of all 2^10^ = 1024 sign patterns, restricted–residual bootstrap–DGP) [48], which do not rely on asymptotic *t* (*G* − 1) approximations and represent a well‐established small‐cluster inference procedure (Table 7). Results corroborate the leave‐one‐cluster‐out pattern: Depersonalization remained statistically supported (exact *p* = 0.003), personal accomplishment remained nonsignificant (exact *p* = 0.226), and emotional exhaustion—significant under the *t* (9) approximation (*p* = 0.028)—did not meet the conventional 0.05 significance threshold under the exact wild bootstrap (*p* = 0.052). We report this discrepancy transparently rather than selecting the more favorable inferential method: Depersonalization is the dimension with the most robust statistical support across every correction applied in this manuscript, while the emotional‐exhaustion association, though directionally consistent throughout, showed less stable inferential support across small‐cluster procedures.

**TABLE 7:** Wild‐cluster‐bootstrap *p*‐values (exact enumeration, 1024 sign patterns) compared with *t* (df = 9) cluster‐robust *p*‐values.

Predictor	*t*‐statistic (CR1)	*p*, *t* (df = 9)	*p*, exact wild bootstrap
Emotional exhaustion	−2.623	0.028	0.052
Depersonalization	−6.970	< 0.001	0.003
Personal accomplishment	−1.428	0.187	0.226

### 5.8. Deviance Statistics and Variance Components

The REML‐based deviance values were 1655.54 for the null model and 1590.63 for the burnout model. Because REML likelihoods are not directly comparable across models with different fixed‐effects specifications, these values are reported descriptively rather than interpreted as a formal test of model‐fit improvement. Between‐unit variance in patient experience decreased from 0.191 to 0.145, corresponding to a proportional reduction of approximately 24.1% relative to the null model, which we interpret as a proportional variance reduction rather than a definitive estimate of explained variance (see Table 8).

**TABLE 8:** Deviance statistics and variance components for the null and burnout models.

Model	−2 log likelihood	Between‐unit variance	Within‐unit variance
Null model	1655.54	0.191	0.643
Burnout model	1590.63	0.145	0.589

*Note:* Between‐unit variance decreased from 0.191 to 0.145, representing a proportional reduction of approximately 24.1% relative to the null model. The REML‐based deviance values are presented descriptively and are not interpreted as a formal likelihood‐based comparison of model fit because the models differ in their fixed‐effects specification.

In practical terms, the observed associations were modest in absolute magnitude. For example, the emotional‐exhaustion coefficient (*B* = −0.022) implies that a full 2‐SD difference in unit‐level emotional exhaustion (≈ 4.1 points) is associated with roughly a 0.09‐point difference on the 1–4 HCAHPS composite—noticeable at the unit level but small relative to the total spread of unit means (range 2.18–3.76).

A one‐unit increase in depersonalization was associated with an estimated 0.015‐point decrease in HCAHPS scores. Given that the standard deviation of the corrected HCAHPS composite in the present sample was 0.853 (patient level)/0.475 (unit level), this corresponds to a small effect size in absolute terms, even though it is now the most statistically robust of the three burnout‐dimension associations under cluster‐robust inference.

The null‐model ICC (ICC = 0.229) indicated appreciable between‐unit variation in patient experience, consistent with H4. The subsequent reduction in between‐unit variance from 0.191 to 0.145 following inclusion of the burnout predictors provides a descriptive indication that these predictors account for part of this between‐unit variation within the present sample. Given the modest number of clusters, this proportional reduction is interpreted as evidence of contextual association rather than a precise estimate of variance explained.

Summarizing hypothesis testing: H_1_ (emotional exhaustion) received qualified support, remaining directionally consistent and significant under *t* (9) inference but only borderline under the most conservative exact wild‐cluster bootstrap (*p* = 0.052). H_2_ (depersonalization) received the strongest support, remaining significant across every specification and sensitivity check. H_3_ (personal accomplishment) was not supported: The multivariable coefficient was in the opposite direction to that hypothesized (negative rather than positive) and was not statistically significant (robust *p* = 0.187); this reversal could not be attributed to coding or scale‐direction error and is discussed further below and in the study limitations. H_4_ was supported by the presence of appreciable between‐unit variation in patient experience (ICC = 0.229). The proportional reduction in between‐unit variance following inclusion of the burnout predictors provides additional descriptive evidence that these predictors account for part of this variation.

## 6. Discussion

This study examined the association between nurse burnout and patient‐reported experience across clinical units using a multilevel framework, contributing contextually grounded evidence from a Southeast European hospital setting. Among the three dimensions examined, depersonalization showed the most statistically robust association with patient experience across the inferential and sensitivity analyses, whereas emotional exhaustion showed a directionally consistent but less stable association that became borderline under exact wild‐cluster‐bootstrap inference. Personal accomplishment was not statistically significant. These results suggest a contextual correspondence between unit‐level burnout and patient‐perceived care quality [18, 21]. Given the limited number of clinical units, and the heterogeneous cross‐hospital composition of those clusters (Supporting Table S1), the findings should be interpreted as exploratory contextual associations and not as precise unit‐level estimates [44].

The study does not assume strong within‐unit consensus in burnout perceptions. Instead, unit‐level scores are interpreted as reflecting aggregated contextual strain, that is, average work‐environment pressure within clinical units, rather than homogeneous team climates. This perspective allows examination of whether variation in aggregated strain corresponds with patient‐perceived care quality even when individual nurse experiences vary. In interpreting the relative importance of the burnout dimensions, it is important to distinguish between the extent to which a dimension shows evidence of clustering and the statistical robustness of its regression association with patient experience. Depersonalization provided weaker evidence of within‐unit agreement as a collective construct but showed the most statistically robust association with patient experience across sensitivity analyses. Emotional exhaustion showed clearer evidence of clustering as an aggregated contextual indicator, but its regression coefficient was less stable under conservative small‐cluster corrections.

### 6.1. Emotional Exhaustion and Depersonalization as Contextual Associations

Emotional exhaustion was associated with lower patient‐experience scores. Units characterized by sustained fatigue and emotional strain tended to report less favorable patient perceptions of care, consistent with the JD–R model, which links prolonged job demands to reduced engagement and responsiveness [2, 40]. At a theoretical level, emotional depletion may limit nurses’ capacity to maintain attentive communication and emotional support across a shift, particularly in high‐intensity units. Even when clinical tasks are completed effectively, reduced emotional availability may plausibly influence how patients interpret responsiveness and attentiveness in care encounters—though, as noted above, this remains a contextual association rather than an observed individual‐level mechanism in the present data [1, 5].

This pattern can also be interpreted through the lens of emotional labor, which refers to the regulation of emotions required to maintain a caring and responsive professional role during patient interactions [49]. Nursing requires constant attention, empathy, and interpersonal interaction that may be perceptible to patients. Psychological tiredness can impair emotional labor, potentially contributing to small but noticeable changes in tone, attentiveness, and emotional availability during care exchanges. These findings are also conceptually consistent with relational coordination theory, which stresses communication quality, shared goals, and mutual respect in care experiences [50]. Emotional exhaustion may be associated with these social processes by lowering interpersonal involvement, although the present study did not directly measure these mechanisms. Depersonalization may be even more directly relevant to patient‐facing relational processes because it explicitly reflects interpersonal distancing; indeed, its association with patient experience was more statistically robust than that of emotional exhaustion in the present analyses. Personal accomplishment may indicate an important psychological or professional aspect of burnout, although its manifestations may be less directly observable in short‐term patient‐facing exchanges.

Across model specifications, depersonalization remained statistically significant under every correction applied—cluster‐robust *t* (9) inference (*p* < 0.001), leave‐one‐cluster‐out reestimation (*p* < 0.01 in all 10 exclusions), and exact wild‐cluster‐bootstrap inference (*p* = 0.003)—establishing it as the most robust unit‐level contextual indicator in this study (see Figure 2). Emotional exhaustion was directionally consistent throughout but was more fragile: significant under *t* (9) (*p* = 0.028) and in 6 of 10 leave‐one‐cluster‐out exclusions (Supporting Table S5), but only borderline under the more conservative exact wild‐cluster bootstrap (*p* = 0.052; Table 7). Personal accomplishment did not reach significance under any specification. The two dimensions were themselves strongly intercorrelated at the unit level (*r* = 0.87), consistent with Maslach’s multidimensional burnout theory but also indicating that, in this sample, they may substantially capture overlapping contextual strain rather than fully distinct constructs; unit‐level VIFs of 6.2 (EE) and 8.9 (DP) confirm this collinearity is nontrivial (Supporting Table S3) and likely contributes to emotional exhaustion’s comparative fragility once depersonalization is held constant in the same model. Given this strong unit‐level intercorrelation and the small number of Level‐2 clusters, the mutually adjusted EE and DP coefficients should not be interpreted as fully independent dimension‐specific effects. Rather, they indicate overlapping contextual associations whose relative statistical stability differs across specifications. From a theoretical perspective, this pattern is broadly consistent with Maslach’s multidimensional burnout theory and the JD–R model in a multilevel framework: Under prolonged job demands, sustained emotional and interpersonal strain may plausibly affect patient communication, attentiveness, and responsiveness, a process‐level expression of contextual strain consistent with the Donabedian structure–process–outcome model. This interpretation is further illustrated in the simplified interpretative pathway presented in Figure 2 and should be read as a theoretical proposal motivated by the contextual (unit‐level) pattern rather than as a confirmed individual‐level causal pathway.

### 6.2. Depersonalization and Relational Aspects of Care

Among the burnout dimensions, depersonalization showed the strongest coefficient in the initial conventional specification and, following correction of the composite‐scoring error and application of small‐cluster (df = 9) inference, it was also the most statistically robust dimension under cluster‐robust estimation (robust *p* < 0.001). Clinical units in which nurses reported higher levels of emotional distancing and reduced empathy tended to show lower HCAHPS scores, suggesting that patients’ perceptions of care may be particularly sensitive to unit‐level patterns of relational strain. This pattern aligns with Maslach’s conceptualization of burnout, which identifies depersonalization as the dimension most directly connected to interpersonal interaction and communication with patients [7, 24].

Depersonalization may theoretically manifest through briefer communication, diminished attentiveness, or reduced empathic engagement. In high‐demand hospital environments, emotional distancing can function as a coping strategy that sustains task performance under pressure while altering the relational tone of care. International research similarly links nurse detachment and missed care to lower patient satisfaction and less favorable care evaluations [21, 51], highlighting the vulnerability of relational aspects of care to burnout‐related strain. However, these potential behavioral mechanisms were not directly measured in the present study and should therefore be regarded as theoretical interpretations rather than demonstrated pathways.

Its association remained statistically significant under cluster‐robust, small‐cluster‐corrected estimation, even though aggregation diagnostics (Table 6) showed limited within‐unit agreement (mean rwg = −0.46) and minimal ICC (1) (0.01) for this dimension. This combination is worth flagging explicitly: A robust unit‐level regression association can coexist with weak evidence that individual nurses within a unit actually agree on their depersonalization levels. We interpret this as meaning the unit‐level mean of depersonalization is statistically associated with patient experience, without implying that depersonalization functions as a shared, agreed‐upon climate within units. Depersonalization may instead reflect concurrent, nonconsensual individual strain that nonetheless aggregates into a meaningful unit‐level signal. Given the strong intercorrelation with emotional exhaustion (*r* = 0.87), the depersonalization coefficient should not be interpreted as a fully independent effect distinct from emotional exhaustion. Nevertheless, its persistence across all leave‐one‐cluster‐out analyses and the exact wild‐cluster‐bootstrap procedure indicates that it represents the statistically most stable contextual association in the present dataset. We therefore regard this finding as important but exploratory, pending replication in studies with a larger number of more homogeneously defined clusters.

### 6.3. Personal Accomplishment and Professional Engagement

In the bivariate unit‐level correlation (Table 2), higher personal accomplishment showed a negligible positive association with patient experience (*r* = 0.04), broadly consistent with the theoretical expectation that greater professional efficacy corresponds with more favorable nurse‐patient interaction [18, 26]. In the multivariable multilevel model, however, the personal‐accomplishment coefficient was negative (*B* = −0.019, robust *p* = 0.187, not statistically significant), meaning that once emotional exhaustion and depersonalization are held constant, units with higher aggregated personal accomplishment show a slightly lower predicted patient‐experience score. This sign reversal between the bivariate and multivariable results was investigated and is not attributable to a coding or scale‐direction error: item‐total correlations within the personal‐accomplishment subscale are positive in the expected direction, and personal accomplishment correlates with emotional exhaustion and depersonalization in the theoretically expected directions (Table 2). We therefore treat the negative multivariable coefficient as an observed but statistically nonsignificant pattern that may reflect statistical suppression arising from shared variance among the burnout dimensions. Given the small number of Level‐2 units and the substantial collinearity between emotional exhaustion and depersonalization, however, the mechanism underlying this sign reversal cannot be established from the present data. This finding should be regarded as unresolved and examined in future studies with larger cluster counts and reduced collinearity among the burnout dimensions.

This dimension’s association did not reach statistical significance under either the cluster‐robust, small‐cluster‐corrected estimation or the additional sensitivity analyses. Accordingly, no directional unit‐level association between personal accomplishment and patient experience can be established from the present data. Personal accomplishment should therefore be interpreted cautiously as an exploratory indicator of aggregated contextual strain rather than as a confirmed protective or risk factor at the unit level. The unresolved sign reversal further supports treating this dimension as exploratory pending replication.

### 6.4. Unit‐Level Variation and Contextual Influences

The multilevel analysis revealed meaningful heterogeneity in patient experience across clinical units, with 22.9% of the total variance attributable to between‐unit differences (ICC = 0.229). The reduction of approximately 24.1% in between‐unit variance after inclusion of burnout dimensions indicates that the burnout predictors statistically account for a portion of the observed between‐unit variation in patient experience within this sample. This proportional reduction should not be interpreted as evidence that burnout causally explains 24.1% of between‐unit differences, particularly given the limited number of clusters and the cross‐hospital heterogeneity documented in Supporting Table S1.

These findings are consistent with multilevel perspectives on healthcare quality and with Donabedian’s structure–process–outcome framework, in which structural and organizational conditions influence care processes and, ultimately, patient‐reported outcomes [35, 41]. Within this framework, burnout indicators are best understood as markers of aggregated contextual strain rather than direct causal mechanisms or cohesive climate constructs.

Because staffing adequacy, workload intensity, leadership climate, and patient case‐mix were not directly measured, the observed associations may reflect cumulative organizational demands embedded within specific units. Differences in patient complexity across units may also contribute to variation in patient experience. Burnout is therefore interpreted as a contextual proxy of work‐environment pressure rather than a causal unit‐level climate. Furthermore, grouping functionally similar clinical units across different hospitals may obscure hospital‐specific organizational influences and increase within‐cluster heterogeneity. Accordingly, the resulting estimates should be interpreted as associations across analytically constructed functional clusters rather than effects attributable to coherent hospital‐specific clinical units.

These results are consistent with recent large‐scale evidence. A 2024 meta‐analysis including over 280,000 nurses reported that burnout is associated with lower patient satisfaction, reduced quality of care, and poorer patient safety; as this evidence is based on observational data, it supports a contextual rather than causal interpretation of the present findings [18].

Beyond statistical significance, the practical magnitude of these associations is modest. A full 2‐SD difference in unit‐level emotional exhaustion (≈ 4.1 points on the MBI subscale) is associated with roughly a 0.09‐point difference on the 1–4 HCAHPS composite, and a comparable calculation for depersonalization yields a similarly small absolute shift. These are nontrivial differences at the unit level—enough to distinguish better‐ from worse‐performing units in relative terms—but they are small relative to the full 1–4 range of the instrument and should not be overinterpreted as implying large changes in individual patients’ care experience. We report them because contextual associations of this size can still be meaningful for identifying units warranting closer attention, not because they represent large effects in absolute terms.

### 6.5. Implications for Practice and Policy

The findings suggest that nurse well‐being may be relevant to patient‐perceived quality of care and support further attention to workforce conditions as a component of healthcare quality. However, given the observational design and the exploratory nature of the unit‐level estimates, the present findings should not be interpreted as evidence that specific organizational interventions will necessarily improve patient experience. Organizational strategies that improve working conditions and reduce excessive workload may therefore contribute to more responsive and patient‐centered care environments. At the unit level, these findings support the implementation of structured burnout monitoring systems, such as periodic assessment of emotional exhaustion using validated instruments, to identify early signs of contextual strain. Team‐based management approaches, including workload redistribution, supportive supervision, and communication‐focused interventions, may help mitigate the impact of sustained job demands on care delivery. Strategies addressing staffing adequacy, leadership support, team functioning, and excessive workload represent plausible organizational targets that warrant prospective evaluation. Periodic assessment of nurse well‐being may also help organizations identify patterns of workforce strain, although the present study does not establish that burnout monitoring itself improves patient outcomes.

These results highlight the relevance of unit‐level organizational interventions, as evidence suggests that system‐level changes can yield broader workforce and care‐quality benefits [52, 53]. Given that depersonalization showed the most statistically robust association while emotional exhaustion showed a directionally consistent but more fragile pattern, the present findings do not support prioritizing one burnout dimension as a definitive intervention target. Rather, they support broader investigation of organizational conditions that may contribute to aggregated emotional and interpersonal strain within clinical units.

### 6.6. Future Research

Future research should build on these findings using longitudinal designs to examine how changes in burnout over time relate to patient‐experience outcomes. Intervention‐based studies evaluating staffing adjustments, leadership development, or well‐being programs would help clarify whether improvements in working conditions translate into measurable changes in patient experience. Incorporating additional organizational indicators such as staffing ratios, workload intensity, and leadership climate may further clarify the contextual factors shaping aggregated contextual strain within clinical units. Mixed‐method approaches combining quantitative and qualitative data could deepen understanding of how unit climate and teamwork influence patient perceptions. Larger multilevel samples including more clinical units will be essential for strengthening contextual inference and enabling more precise estimation of unit‐level effects. Future studies should preferentially preserve hospital‐specific clinical units as the Level‐2 organizational structure and include sufficient numbers of such units to permit simultaneous modeling of hospital‐ and unit‐level influences. Replication should also assess whether the apparent statistical robustness of depersonalization persists when emotional exhaustion and depersonalization can be estimated with less collinearity and a substantially larger Level‐2 sample.

## 7. Study Limitations

Several limitations should be considered when interpreting these findings, and they define the appropriate scope of inference from the present study.

First, the cross‐sectional design limits the ability to draw causal conclusions about the relationship between nurse burnout and patient experience. While the multilevel analysis identified significant associations at the unit level, the temporal direction of these relationships cannot be definitively established. It remains possible that demanding patient care environments contribute to elevated staff strain, suggesting a potentially reciprocal dynamic that would benefit from longitudinal investigation.

Second, burnout was aggregated at the clinical‐unit level to capture aggregated contextual strain related to shared working conditions rather than to assume homogeneous perceptions within units. This approach aligns with the study’s conceptual framework and allows examination of average contextual strain levels observable within units; nevertheless, aggregation may obscure individual variation among nurses and patients within the same unit. Unit‐level burnout indicators should therefore be interpreted as approximate markers of aggregated contextual strain rather than cohesive shared climate constructs. Depersonalization, in particular, showed minimal between‐unit variance and limited within‐unit agreement, suggesting that it reflects overlapping individual strain rather than a stable collective climate. However, despite this weaker evidence of clustering as a collective construct, the association between unit‐level depersonalization and patient experience was the most statistically robust across the primary model, leave‐one‐cluster‐out analyses, and exact wild‐cluster‐bootstrap inference. Thus, the robustness of the depersonalization coefficient should be distinguished from the strength of evidence that depersonalization represents a cohesive unit‐level climate. Findings related to depersonalization should therefore be interpreted as robust contextual associations in the present dataset, but not as evidence of a stable collective unit‐level construct. Emotional exhaustion showed clearer evidence of clustering as an aggregated contextual indicator; however, its regression association with patient experience was less statistically stable under small‐cluster sensitivity corrections, becoming borderline in the exact wild‐cluster‐bootstrap analysis. Accordingly, stronger clustering of emotional exhaustion should not be interpreted as stronger statistical robustness of its association with patient experience.

Building on this, an additional interpretive limitation should be considered when examining the differential stability of burnout dimensions across analyses. While depersonalization and reduced personal accomplishment are theoretically important components of burnout, their associations with patient experience may not be directly observable within patient‐reported measures such as HCAHPS. These dimensions may operate through more subtle, indirect, or longer term relational and psychological processes that are not immediately perceived by patients during relatively short hospital stays. Accordingly, differences in the observed strength or stability of associations across burnout dimensions should not be interpreted as evidence that one dimension is inherently more or less important clinically, but rather as reflecting both measurement characteristics and the limited number of analytical clusters.

Third, although the sample included a large number of nurses and patients, the number of clinical units available for the multilevel analysis was relatively modest, and—as documented in Supporting Table S1—the 10 analytical clusters varied considerably in how many hospitals they pooled together, from a single hospital to all 11. This heterogeneity means the clusters do not represent a uniformly comparable level of shared contextual environment, and disaggregating by hospital‐specific unit was considered but not adopted because it would have produced many very small clusters. Methodological research indicates that multilevel models based on very few, heterogeneously composed higher level clusters may yield less stable standard errors and imprecise contextual estimates [44, 45], with simulation evidence also suggesting an increased risk of Type I error under such conditions [46]. Although cluster‐robust standard errors with small‐cluster (df = 9) correction were used and leave‐one‐cluster‐out sensitivity analysis was conducted (Supporting Table S5) to mitigate this limitation, statistical stability for the emotional‐exhaustion association varied across exclusions. The exact wild‐cluster‐bootstrap analysis further showed that emotional exhaustion was only borderline statistically significant, whereas depersonalization remained statistically robust. Unit‐level findings are therefore interpreted as exploratory associations reflecting aggregated contextual strain rather than confirmatory contextual effects. Thus, contextual estimates should be considered directionally informative rather than definitive, and future studies employing larger numbers of more homogeneously defined clusters and alternative small‐sample corrections are warranted.

Fourth, although selected patient‐level variables (age and length of stay) were examined in preliminary analyses, a comprehensive case‐mix adjustment was not feasible due to the absence of key clinical variables such as diagnosis, disease severity, or overall health status. As a result, the models may not fully account for all patient‐level differences, and part of the observed association between unit‐level burnout and patient experience may reflect residual confounding. Findings should therefore be interpreted as contextual associations rather than fully adjusted estimates.

Fifth, the study was conducted in public hospitals across a single country. Organizational structures, workforce configurations, and resource availability vary across healthcare systems, which may have an impact on both burnout and patient experience. The transferability of findings to other healthcare contexts, particularly private or differently resourced systems, should therefore be considered carefully.

Sixth, several patient‐experience items were conditionally administered (e.g., medication communication and assistance‐related questions), resulting in structurally missing responses for patients to whom these items did not apply; full documentation is provided in Supporting Table S2 (item 12 only, 6.3% missing, entirely structural). This conditional structure may have contributed to lower internal consistency in domains with a limited number of applicable items, although overall reliability across patient‐experience measures remained acceptable to high (Supporting Table S4).

Seventh, the personal‐accomplishment coefficient showed a sign reversal between the bivariate correlation and the multivariable multilevel model that we could not fully resolve with the present data; we verified it is not a coding error, but the underlying explanation (most likely statistical suppression from collinearity with emotional exhaustion and depersonalization) should be treated as a hypothesis rather than a confirmed mechanism. In addition, the strong correlation between emotional exhaustion and depersonalization at the unit level limits interpretation of their mutually adjusted coefficients as fully independent dimension‐specific effects. Future studies with larger cluster counts and dimension‐specific measurement models are needed to investigate these relationships directly.

Finally, both burnout and patient experience were assessed using validated self‐report instruments. Although these tools are widely used in health services research, responses may be influenced by individual perceptions, expectations, or momentary factors at the time of data collection. The analysis did not include direct structural indicators such as staffing ratios, workload intensity, or leadership climate. Future multilevel studies incorporating these organizational variables would allow a more comprehensive assessment of how aggregated contextual strain and structural conditions jointly relate to patient outcomes.

## 8. Conclusion

This study provides evidence that nurse burnout may extend beyond individual experience and reflect aggregated contextual strain within clinical units associated with patient‐reported care quality. Among the burnout dimensions examined, depersonalization emerged as the most statistically robust contextual indicator linked to patient‐experience scores, remaining significant under every small‐cluster correction applied, including the most conservative exact wild‐cluster‐bootstrap test. Emotional exhaustion showed a directionally consistent but more fragile association, reaching significance under conventional small‐cluster inference but only borderline significance under the most conservative correction. Personal accomplishment did not reach significance under any specification and showed an unresolved sign reversal warranting further study. Units characterized by higher emotional exhaustion and greater depersonalization tended to report less favorable patient‐experience scores, suggesting that the relational tone of care may be perceptible to patients—though this remains a contextual, unit‐level association rather than a demonstrated individual‐level causal mechanism.

Depersonalization showed the strongest and most statistically stable association once the composite‐scoring error was corrected and small‐cluster inference (df = 9) was applied, while emotional exhaustion also remained significant, though somewhat less stable across leave‐one‐cluster‐out checks (Supporting Table S5). Given the limited within‐unit agreement observed for depersonalization (Table 6) and the strong intercorrelation between the two dimensions (*r* = 0.87), these findings warrant replication in a larger and more homogeneously defined set of clusters.

Taken together, these findings suggest that average strain levels within units may become visible in patient‐reported care experiences even when staff perceptions are not fully aligned. The statistical robustness of depersonalization and the directional consistency of emotional exhaustion strengthen the interpretation that sustained team‐level workload pressure may be associated with how care is experienced and evaluated by patients, while the magnitude of these associations remains modest in absolute terms and the number of available clusters remains small.

These findings support further investigation of staffing support, leadership, and team‐level resources as potential organizational targets for improving professional well‐being and patient care experience. Given the exploratory nature of the multilevel estimates, the modest number of clinical units, and variability in within‐unit agreement across burnout dimensions, the findings should be interpreted as associations reflecting aggregated contextual strain rather than definitive unit‐level climate effects. Replication in larger and more diverse healthcare settings with stronger within‐unit clustering and additional structural indicators will be important for confirming and extending these results.

## Author Contributions

Edona Haxhija: conceptualization, data curation, investigation, software, formal analysis, writing–original draft, and writing–review and editing.

Drita Kruja: methodology, validation, formal analysis, project administration, writing–original draft, and writing–review and editing.

## Funding

This research did not receive any specific grant from funding agencies in the public, commercial, or not‐for‐profit sectors.

## Disclosure

The study followed the Strengthening the Reporting of Observational Studies in Epidemiology (STROBE) guidelines. All authors approved the final version of the manuscript and accept responsibility for the integrity and accuracy of the work.

## Ethics Statement

This study was conducted in accordance with the principles of the Declaration of Helsinki and received ethical approval from the Institutional Ethics Committee of the European University of Tirana (approval date: October 07, 2024, Tirana, Albania).

Authorization to collect data in regional public hospitals was granted by the Operator of Health Care Services (Ministry of Health and Social Protection, Republic of Albania) (Protocol No. 3412/1, dated October 15, 2024, Tirana, Albania), permitting data collection in accordance with national legislation on healthcare and personal data protection.

All participants were informed about the purpose of the study, voluntary participation, anonymity, and confidentiality of responses. Verbal informed consent was obtained from all participating nurses and patients before participation. No identifiable personal data were collected, and all information was analyzed in aggregated form to ensure anonymity and ethical compliance.

## Consent

Please see the Ethics Statment.

## Conflicts of Interest

The authors declare no conflicts of interest.

## Supporting Information

Additional supporting information can be found online in the Supporting Information section.

## Supporting information


**Supporting Information 1** Supporting Table S1. Cross‐hospital composition of the 10 analytical clusters. Supporting Table S2. Missing data by item (*N* = 679 patients). Supporting Table S3. Variance inflation factors (VIF) for burnout predictors, computed at the correct (unit) level of analysis vs. the patient level. Supporting Table S4. Domain‐level internal consistency (Cronbach’s alpha) and one‐factor CFA loadings summary. Supporting Table S5. Leave‐one‐cluster‐out sensitivity for all three burnout‐dimension coefficients.

**Supporting Information 2** STROBE Checklist.

## Data Availability

The data supporting the findings of this article are available from the corresponding author (E.H.) upon reasonable request.
