# Supplementary material for: Unit‐Level Burnout Dimensions and Patient Experience: A Multilevel Study of Aggregated Contextual Strain in a Southeast European Health System
Source: Nurs Res Pract. 2026 Oct 25;2026:7626110. doi: 10.1155/nrp/7626110 (PMC13695889; doi:10.1155/nrp/7626110)
Supplement: Supplementary file 1 [file NRP-2026-7626110-s001.docx]

STROBE Statement—Checklist of items that should be included in reports of ***cross-sectional studies***

***Manuscript 7626110- “Unit-Level Burnout Dimensions and Patient Experience: A Multilevel Study of Aggregated Contextual Strain in a Southeast European Health System” (Haxhija & Kruja) - Nursing Research and Practice***

| **Section/ Topic** | **Item No** | **Recommendation** | **Reported on Page No. (manuscript location)** |
| --- | --- | --- | --- |
| **Title and abstract** | 1 | (a) Indicate the study’s design with a commonly used term in the title or the abstract | p. 1 - Title (“…A Multilevel Study of Aggregated Contextual Strain…”); Abstract, Methods sentence: “A cross-sectional multilevel study was conducted across 11 regional public hospitals…” |
|  |  | (b) Provide in the abstract an informative and balanced summary of what was done and what was found | p. 1 - Abstract (Purpose, Methods, Results, Conclusion paragraphs) |
| **Introduction** | | | |
| **Background/ rationale** | 2 | Explain the scientific background and rationale for the investigation being reported | pp. 1- 4- Section 1, Introduction (paras 1-4) and Section 2, Literature Review |
| **Objectives** | 3 | State specific objectives, including any prespecified hypotheses | p. 2 – Introduction, aim statement (“This study examines all three Maslach burnout dimensions…”); p. 6 – Section 3, Theoretical Framework and Hypotheses (H1–H4) |
| **Methods** | | | |
| **Study design** | 4 | Present key elements of study design early in the paper | p. 7 – Section 4.1, Research design, para 1 (“This study employed a cross-sectional multilevel design…”) |
| **Setting** | 5 | Describe the setting, locations, and relevant dates, including periods of recruitment, exposure, follow-up, and data collection | p. 7 – Section 4.1 (11 regional public hospitals, Albania, October 2024–July 2025); p. 9 – Section 4.4, Sampling, para 1 |
| **Participants** | 6 | (a) Give the eligibility criteria, and the sources and methods of selection of participants | p. 9 – Section 4.4, Sampling, paras 2–4 (eligibility/exclusion criteria; total-eligible-population approach for nurses; consecutive sampling for patients before discharge) |
| **Variables** | 7 | Clearly define all outcomes, exposures, predictors, potential confounders, and effect modifiers. Give diagnostic criteria, if applicable | p. 7 – Section 4.2.1, Nurse Burnout (predictors: EE, DP, PA); p. 7 – Section 4.2.2, Patient experience (outcome: HCAHPS composite); p. 8 – Section 4.2.3, Control variables (age, gender, education as candidate confounders) |
| **Data sources/ measurement** | 8* | For each variable of interest, give sources of data and details of methods of assessment (measurement). Describe comparability of assessment methods if there is more than one group | pp. 7–8 – Sections 4.2.1–4.2.2 (MBI and adapted HCAHPS instruments, item counts, response scales, forward-backward translation p. 7, 20-patient wording pilot p. 8); p. 8 – Section 4.3, Questionnaire design (administration mode) |
| **Bias** | 9 | Describe any efforts to address potential sources of bias | p. 7 – forward-backward translation/expert-panel adjudication; p. 9 – Sampling, para 4 (nonresponse pattern checked across hospitals); p. 11 – Section 4.5 (grand-mean centering, unit-level VIF diagnostics); pp. 10–11 and p. 16 – small-cluster t(9) correction and exact wild-cluster-bootstrap (Table 7) |
| **Study size** | 10 | Explain how the study size was arrived at | p. 9 - Section 4.4, Sampling (the numbers of eligible, approached, and included nurses and patients are reported, resulting in a final analytic sample of 2,000 nurses and 679 patients) |
| **Quantitative variables** | 11 | Explain how quantitative variables were handled in the analyses. If applicable, describe which groupings were chosen and why | p. 8 – Section 4.2.2 (0–10 global item linearly rescaled to the 1–4 metric; composite = mean of non-missing 1–4 items); p. 11 – Section 4.5 (unit-level burnout predictors grand-mean centered; all variables retained as continuous, no categorisation) |
| **Statistical methods** | 12 | (a) Describe all statistical methods, including those used to control for confounding | pp. 10–11 – Section 4.5, Data analysis, paras 1–2 (null-model ICC; REML random-intercept multilevel models; burnout dimensions entered simultaneously; cluster-robust CR1 inference) |
|  |  | (b) Describe any methods used to examine subgroups and interactions | p. 11 – Section 4.5 (“Random slopes were not estimated… Cross-level interactions were not specified”) – not performed, due to limited clusters |
|  |  | (c) Explain how missing data were addressed | p. 11 – Section 4.5, para 4 (“pairwise, not listwise”); Supplementary Table S2 (Suppl. p. 1) |
|  |  | (d) If applicable, describe analytical methods taking account of sampling strategy | Not applicable – total-eligible-population/consecutive sampling was used (not a complex/stratified survey design); the multilevel patients-within-units structure is addressed under 12(a) |
|  |  | (e) Describe any sensitivity analyses | p. 15 – Section 5.7, Leave-one-cluster-out sensitivity analysis (Supplementary Table S5, Suppl. p. 2); p. 16 – Table 7, exact wild-cluster-bootstrap; p. 15 – sensitivity models with age/length-of-stay covariates |
| **Results** | | | |
| **Participants** | 13* | (a) Report numbers of individuals at each stage of study — eg numbers potentially eligible, examined for eligibility, confirmed eligible, included in the study, completing follow-up, and analysed | p. 9 – Section 4.4, paras 3–4 and Table 1 (2,633 → 2,000 nurses; 1,026 → 679 patients); p. 10 – Figure 3, participant flow diagram |
|  |  | (b) Give reasons for non-participation at each stage | p. 9 – Section 4.4, para 3–4 (nurse exclusions: incomplete MBI, non-inpatient role; patient exclusions: refusal n=200, ineligibility n=47, other n=100) |
|  |  | (c) Consider use of a flow diagram | p. 10 – Figure 3, Participant flow diagram for nurses and patients |
| **Descriptive data** | 14* | (a) Give characteristics of study participants (eg demographic, clinical, social) and information on exposures and potential confounders | p. 9 – Table 1 (nurse/patient counts by hospital); p. 12 – Table 2 (descriptive statistics of unit-level burnout dimensions and patient experience, n = 10 clusters) |
|  |  | (b) Indicate number of participants with missing data for each variable of interest | Supplementary Table S2, Missing data by item (Suppl. p. 1) – only item 12 affected (6.3%) |
| **Outcome data** | 15* | Report numbers of outcome events or summary measures | p. 12 – Section 5.1 and Table 2: patient-level mean HCAHPS (M = 2.572, SD = 0.853, N = 679) and unit-level mean HCAHPS (M = 2.702, SD = 0.475, n = 10) |
| **Main results** | 16 | (a) Give unadjusted estimates and, if applicable, confounder-adjusted estimates and their precision (eg, 95% confidence interval). Make clear which confounders were adjusted for and why they were included | p. 13 – Table 3 (unadjusted unit-level Pearson correlations); p. 15 – Table 6 (mutually adjusted multilevel model, model-based SE and cluster-robust 95% CI, t(9)) and Section 5.6 narrative |
|  |  | (b) Report category boundaries when continuous variables were categorized | Not applicable – burnout dimensions and the HCAHPS composite were retained as continuous variables throughout |
|  |  | (c) If relevant, consider translating estimates of relative risk into absolute risk for a meaningful time period | p. 16 – Section 5.8, “In practical terms…” paragraph; p. 20 – Discussion, “Beyond statistical significance…” paragraph (coefficients translated into absolute HCAHPS-point differences for a 2-SD change in EE/DP) |
| **Other analyses** | 17 | Report other analyses done — eg analyses of subgroups and interactions, and sensitivity analyses | p. 15 – Section 5.7 and Supplementary Table S5 (leave-one-cluster-out, all three dimensions, Suppl. p. 2); p. 16 – Table 7 (exact wild-cluster-bootstrap); p. 15 – Section 4.5 (age/length-of-stay covariate check) |
| **Discussion** | | | |
| **Key results** | 18 | Summarise key results with reference to study objectives | p. 17 – Section 6, Discussion, opening paragraphs (before 6.1); p. 23 – Section 8, Conclusion, para 1 |
| **Limitations** | 19 | Discuss limitations of the study, taking into account sources of potential bias or imprecision. Discuss both direction and magnitude of any potential bias | pp. 21–24 – Section 7, Study limitations (eight numbered limitations: causality/cross-sectional design; aggregation and within-unit heterogeneity; small/heterogeneous cluster count; residual confounding; single-country setting; conditional-item missingness; PA sign reversal/collinearity; self-report measurement) |
| **Interpretation** | 20 | Give a cautious overall interpretation of results considering objectives, limitations, multiplicity of analyses, results from similar studies, and other relevant evidence | pp. 17–22 – Section 6, Discussion (subsections 6.1–6.6, consistently using associational rather than causal language); pp. 23–24 – Section 8, Conclusion |
| **Generalisability** | 21 | Discuss the generalisability (external validity) of the study results | p. 23 – Section 7, Study limitations, “Fifth” paragraph (transferability to private/differently resourced health systems); p. 2 – Introduction, para 4 (cross-national applicability caveat) |
| **Other information** | | | |
| **Funding** | 22 | Give the source of funding and the role of the funders for the present study and, if applicable, for the original study on which the present article is based | p. 25 – end-matter “Funding” statement: “This research did not receive any specific grant from funding agencies in the public, commercial, or not-for-profit sectors.” |

*Give information separately for exposed and unexposed groups.

**Note:** An Explanation and Elaboration article discusses each checklist item and gives methodological background and published examples of transparent reporting. The STROBE checklist is best used in conjunction with this article (freely available on the Web sites of PLoS Medicine at http://www.plosmedicine.org/, Annals of Internal Medicine at http://www.annals.org/, and Epidemiology at http://www.epidem.com/). Information on the STROBE Initiative is available at www.strobe-statement.org.
