# Supplementary material for: Unit‐Level Burnout Dimensions and Patient Experience: A Multilevel Study of Aggregated Contextual Strain in a Southeast European Health System
Source: Nurs Res Pract. 2026 Oct 25;2026:7626110. doi: 10.1155/nrp/7626110 (PMC13695889; doi:10.1155/nrp/7626110)
Supplement: Supplementary file 2 [file NRP-2026-7626110-s002.docx]

**Supplementary Materials**

**Unit-Level Burnout Dimensions and Patient Experience: A Multilevel Study of Aggregated Contextual Strain in a Southeast European Health System**

This file contains supplementary tables referenced in the main manuscript. These tables provide additional transparency, diagnostic, and sensitivity-analysis detail supporting the main results reported in Tables 1-8 of the manuscript.

**Supplementary Table S1. Cross-hospital composition of the 10 analytical clusters**

To assess the comparability of the 10 analytical clusters used in the multilevel models, this table documents the number of distinct hospitals contributing patients to each cluster. Clusters range from drawing on a single hospital (cluster 16, n = 21 patients, 1 hospital) to pooling patients from all 11 hospitals (clusters 1 and 4). This heterogeneity means the 10 clusters do not represent a uniformly comparable level of cross-hospital aggregation and is discussed as a limitation of the clustering approach in the main manuscript (Section 4.4 and Study Limitations).

| **Cluster (Unit)** | **Hospitals contributing (Hospital ID)** | **N hospitals** | **N patients** |
| --- | --- | --- | --- |
| 1 | 1,2,3,4,5,6,7,8,9,10,11 | 11 | 99 |
| 2 | 1,2,3,4,5,6,7,8,9,11 | 10 | 105 |
| 3 | 1,2,4,5,6,7,8,10,11 | 9 | 69 |
| 4 | 1,2,3,4,5,6,7,8,9,10,11 | 11 | 141 |
| 5 | 1,2,3,4,5,6,7,8,9,10 | 10 | 75 |
| 8 | 1,3,5,6,7,8 | 6 | 61 |
| 9 | 1,5,6,7,8 | 5 | 47 |
| 10 | 1,4,6,7,9 | 5 | 42 |
| 11 | 1,3,5,11 | 4 | 19 |
| 16 | 1 | 1 | 21 |

**Supplementary Table S2. Missing data by item (N = 679 patients)**

Only one item across the entire 17-item questionnaire had any missing responses: item 12 ("before giving you any new medicine, how often did staff tell you what the medicine was for"), which is structurally conditional on item 11 ("were you given any medicine you had not taken before"). All other items, and the corrected composite score itself, had 0% missingness.

| **Item** | **N missing** | **% missing** | **Missingness type** |
| --- | --- | --- | --- |
| Item 12 (informed of new-medicine purpose) | 43 | 6.3% | Structural (conditional on Item 11 = "no new medicine given") |
| All other items (1-11, 13-17) and composite score | 0 | 0.0% | — |

**Supplementary Table S3. Variance inflation factors (VIF) for burnout predictors, computed at the correct (unit) level of analysis vs. the patient level**

Variance inflation factors computed at the patient level (n = 679, treating each patient as an independent observation) were below 3. However, because emotional exhaustion, depersonalization, and personal accomplishment are level-2 (unit-level) predictors that are constant within each cluster, patient-level VIFs substantially understate the true collinearity at the level at which these coefficients are actually identified. Recomputed at the unit level (n = 10, the correct level of analysis for these predictors), VIFs confirm non-trivial collinearity between emotional exhaustion and depersonalization, consistent with their r = .87 intercorrelation (Table 3 in the main manuscript).

| **Predictor** | **VIF (patient-level, n=679; incorrect)** | **VIF (unit-level, n=10; correct)** |
| --- | --- | --- |
| Emotional exhaustion | 1.41 | 6.17 |
| Depersonalization | 1.24 | 8.95 |
| Personal accomplishment | 1.22 | 2.38 |

**Supplementary Table S4. Domain-level internal consistency (Cronbach's alpha) and one-factor CFA loadings summary**

Domain-level Cronbach's alphas (raw 1-4 items) ranged from .63 (responsiveness of hospital staff, 2 items) to .99 (medication communication, corrected formula). Given the dominant single-factor structure reported in the main manuscript (Section 5.3), the overall composite score is used as the primary outcome throughout the Results, while the domain structure is presented descriptively rather than as evidence of separate validated subscales.

| **Domain / item pool** | **Cronbach's alpha** | **CFA loading range (1-factor model)** |
| --- | --- | --- |
| Nurse communication (items 1-3) | .97 | .79-.83 |
| Physician communication (items 4-6) | .97 | .82-.85 |
| Responsiveness of staff (items 7-8) | .63 | .74-.75 |
| Pain management (items 9-10) | .93 | .86-.87 |
| Medication communication (items 12-13, corrected) | .99 | .80-.82 |
| Hospital environment (items 14-15) | .87 | .78-.81 |
| Full 16-item composite | .94 | .71-.87 |

**Supplementary Table S5. Leave-one-cluster-out sensitivity for all three burnout-dimension coefficients**

Given the modest number of level-2 clusters (n = 10), all three burnout-dimension coefficients were re-estimated 10 times, each time excluding one cluster, with cluster-robust standard errors and t(df = 8) inference. Depersonalization was the most stable, remaining statistically significant (p < .01) in all 10 exclusions. Emotional exhaustion remained negative and of similar magnitude in every exclusion (range: -0.015 to -0.026) but crossed the p < .05 threshold in only 6 of 10 exclusions. Personal accomplishment was never statistically significant in any exclusion. This pattern, together with the exact wild-cluster-bootstrap results reported in Table 7 of the main manuscript, reinforces depersonalization as the more robust unit-level signal once small-cluster fragility is taken into account.

| **Cluster excl.** | **B (EE)** | **p (EE)** | **B (DP)** | **p (DP)** | **B (PA)** | **p (PA)** |
| --- | --- | --- | --- | --- | --- | --- |
| 1 | -0.0200 | .105 | -0.0134 | <.001 | -0.0174 | .332 |
| 2 | -0.0188 | .067 | -0.0150 | <.001 | -0.0250 | .081 |
| 3 | -0.0153 | .071 | -0.0151 | .001 | -0.0110 | .411 |
| 4 | -0.0246 | .026 | -0.0154 | .001 | -0.0221 | .146 |
| 5 | -0.0214 | .068 | -0.0130 | .001 | -0.0110 | .482 |
| 8 | -0.0255 | .027 | -0.0153 | <.001 | -0.0171 | .242 |
| 9 | -0.0234 | .027 | -0.0152 | <.001 | -0.0173 | .270 |
| 10 | -0.0255 | .014 | -0.0150 | <.001 | -0.0246 | .083 |
| 11 | -0.0238 | .023 | -0.0144 | <.001 | -0.0222 | .144 |
| 16 | -0.0215 | .032 | -0.0143 | .001 | -0.0165 | .255 |
